# Editorial: Novel approaches to the treatment of multidrug-resistant bacteria, Volume II

**DOI:** 10.3389/fphar.2022.1090618

**Published:** 2022-11-30

**Authors:** Priyia Pusparajah, Vengadesh Letchumanan, Bey Hing Goh, Lyndy Joy McGaw

**Affiliations:** ^1^ Medical Health and Translational Research Group (MHTR), Microbiome and Bioresource Research Strength (MBRS), Jeffrey Cheah School of Medicine and Health Sciences, Monash University Malaysia, Bandar Sunway, Selangor Darul Ehsan, Malaysia; ^2^ Novel Bacteria and Drug Discovery Research Group (NBDD), Microbiome and Bioresource Research Strength (MBRS), Jeffrey Cheah School of Medicine and Health Sciences, Monash University Malaysia, Bandar Sunway, Selangor Darul Ehsan, Malaysia; ^3^ Biofunctional Molecule Exploratory (BMEX) Research Group, School of Pharmacy, Monash University Malaysia, Bandar Sunway, Selangor Darul Ehsan, Malaysia; ^4^ College of Pharmaceutical Sciences, Zhejiang University, Hangzhou, China; ^5^ Phytomedicine Programme, Department of Paraclinical Sciences, Faculty of Veterinary Science, University of Pretoria, Pretoria, South Africa

**Keywords:** novel approaches, bacteria, antibacterial, multidrug resistance, biofilm

Overall, the articles in this Research Topic highlight a spectrum of research encompassing the importance of pursuing work to explore new ways of treating multidrug resistant bacteria. Despite the availability of a range of antimicrobials, multidrug resistant (MDR) bacterial infections continue to have a significant impact, contributing to the burden on the public health sector of various countries (Cerceo et al., 2016; Jabbour et al., 2020; Jernigan et al., 2020; Karaman et al., 2020; Kern and Rieg, 2020; Jean et al., 2022). The discovery of antibiotics, starting with the introduction of penicillin in the 1940s, dramatically improved outcomes of bacterial infections, however increasing incidence of infections with multidrug resistant organisms has created an impending healthcare crisis associated with escalating patient morbidity, mortality and health care costs (Medina and Pieper, 2016; Campanini-Salinas et al., 2018; Escolà-Vergé et al., 2020). Traditionally, infections caused by MDR organisms were limited almost exclusively to healthcare associated infections, but today these organisms have spread into the community, stoking fears that we may be entering an era resembling the pre-antibiotic era (Oneko et al., 2015; Monaco et al., 2017; Aston and Wootton, 2020; van Duin and Paterson, 2020; Covington and Rufe, 2021; Guclu et al., 2021; Madrazo et al., 2021). Seeking to find solutions to this predicament requires a multi-pronged approach which will necessitate a deeper understanding of the burden of disease, deciphering the modes of resistance and of course seeking new substances with antibacterial properties as well as exploring repurposing of existing drugs and new drug combinations. In this Research Topic, we explore each of these facets through a range of intriguing articles.

The burden of MDR organisms on the healthcare system in Thailand is clearly illustrated by Tangsawad et al. who reported the results of a retrospective review of the incidence of carbapenem resistant Enterobacterales in Siriraj Hospital, Bangkok, Thailand between January 2015 and December 2019. The data from this retrospective cohort study found 420 cases of CRE over this period of time, with 90.48% of these being carbapenem-resistant *Klebsiella pneumoniae*; 26.9% of the patients were critically ill with the median length of hospitalization being 31 days, and the all-cause in-hospital mortality rate was 68.33%. The median hospitalization cost per admission was US$10,435, giving a strong indication of the burden placed on society by multidrug resistant organisms and highlighting how important it is to begin to take concrete steps to address this problem.

One of the key virulence factors mediating antibiotic resistance in Gram-negative bacteria are resistance-nodulation-division (RND) multidrug efflux pumps (Fernando and Kumar, 2013). Contreras-Gomez et al. sought to decipher the role these pumps play in mediating resistance of the subsets of carbapenem resistant organisms which are also resistant to drug combinations used to target these organisms. They studied the role of these efflux systems on the baseline susceptibility to ceftazidime/avibactam (CZA) and ceftolozane/tazobactam (C/T) in clinical isolates of non-carbapenemase-producing carbapenem-resistant *Pseudomonas aeruginosa* and re-evaluated this in the presence of an RND pump inhibitor. Their conclusion was that RND efflux pumps do participate in baseline resistance to CZA and even more to C/T; however, the genomic diversity of the clinical isolates requires more in-depth analysis to determine the elements directly involved.

In two separate articles, Narayan Pant and his team explore the antibacterial and antibiofilm properties of savirin (*Staphylococcus aureus* virulence inhibitor) (Pant et al.) and ticagrelor (Pant et al.) against *Staphylococcus aureus* in prosthetic joint infections (PJIs), which represents a significant clinical concern. Savirin is a low molecular weight, lipophilic, synthetic molecule, while ticagrelor is a P2Y12 platelet inhibitor. Their work was able to show that in an animal model of biofilm-related *S. aureus* PJI, both these substances, alone and also in combination with cefazolin, were able to reduce bacterial concentrations on implants compared with controls. Ticagrelor and savirin both also reduced bacterial dissemination to periprosthetic tissue. Savirin also downregulated several key biofilm related genes. These results clearly demonstrate the potential of these agents to be potentially viable adjuvants to conventional antibiotics to improve outcomes of prosthetic joint surgery.

Huang et al. explored the ability of a new compound, 666–15, to enhance the activity of polymyxin-B (PB) against *Klebsiella pneumoniae* both *in vitro* and *in vivo* (Huang et al.). Compound 666–15, was originally synthesized in an effort to create potent cyclic adenosine monophosphate response element-binding protein (CREB) inhibitors (Xie et al., 2015), and was part of a screening program by Wei and team to identify PB synergists. Mechanistic studies showed that 666–15 reduced bacterial lipid A modification levels by inhibiting the activity of CrrB. This compound has no antimicrobial activity on its own but was able to enhance the activity of PB against *K. pneumoniae* by inhibiting the CrrA/B TCS. This compound appears to be one of the first inhibitors targeting CrrB which may open new avenues to develop PB synergists by targeting the factors that regulate lipid A modification.

Exploration of traditional medications was carried out by Fan et al. who explored the efficacy of Kangfuxiaoyuan suspension (KFXYs), a traditional Chinese medicine formulation, in treating pelvic inflammatory disease (PID) in a rat model (Fan et al.). PID was induced using a bacterial suspension containing *Staphylococcus aureus* and *Escherichia coli*, and the results of the work demonstrated that KFXYs lowered the rectal temperature and white blood cell counts in acute illness as well as alleviating uterine inflammatory cell infiltration. The KFXYs also significantly improved the effectiveness of levofloxacin. KFXYs is believed to have mediated these effects through inhibition of NF-κB activation by decreasing phosphorylation of p65, demonstrating the potential value of exploring traditional remedies with modern methods.

Given the urgent need to discover new drugs and therapeutics that may provide us with a lifeline to treat these problematic organisms, and the fact that combinations of drugs may yield better results, a new problem for researchers can be trying to select the optimal combination of drugs to experiment with. Lv et al. propose a novel solution through the creation of their Antibiotic Combination DataBase (ACDB) which represents an easily explored resource of current information available about existing antibiotics, providing a means to simplify the process of predicting successful antibiotic combinations (Lv et al.).

In summary, this Research Topic has brought together a spectrum of research highlighting the importance of pursuing work to explore new ways of treating multidrug resistant bacteria; a crucial effort now given the increasing recognition of multidrug resistant organisms as an impending healthcare crisis, even having been highlighted as such by the WHO. The work highlighted here provides hope that there are as yet undiscovered mechanisms that may provide novel means to overcome these MDR organisms. Although a true solution to this will go beyond the laboratory, requiring input and effort in healthcare policy and patient education to reduce indiscriminate antibiotic usage, as well as improved prophylactic strategies, the ongoing research on a global scale is key to providing hope that we can overcome this crisis.
